# Targeting of promising transmembrane proteins for diagnosis and treatment of pancreatic ductal adenocarcinoma

**DOI:** 10.7150/thno.60350

**Published:** 2021-08-25

**Authors:** Vida Mashayekhi, Orsola Mocellin, Marcel H.A.M. Fens, Gerard C. Krijger, Lodewijk A.A. Brosens, Sabrina Oliveira

**Affiliations:** 1 Cell Biology, Neurobiology and Biophysics, Department of Biology, Faculty of Science, Utrecht University, 3584 CH Utrecht, the Netherlands.; 2 Pharmaceutics, Department of Pharmaceutical Sciences, Faculty of Science, Utrecht University, 3584 CG Utrecht, the Netherlands.; 3 Department of Radiology and Nuclear Medicine, University Medical Center Utrecht, Faculty of Medicine, Utrecht University, 3584 CX Utrecht, the Netherlands.; 4 Department of Pathology, University Medical Center Utrecht, Faculty of Medicine, Utrecht University, 3584 CX Utrecht, the Netherlands.

**Keywords:** PDAC, transmembrane proteins, targeted therapy, diagnosis

## Abstract

Pancreatic ductal adenocarcinoma (PDAC) is one of the most fatal types of cancer due to the relatively late diagnosis and the limited therapeutic options. Current treatment regimens mainly comprise the cytotoxic agents gemcitabine and FOLFIRINOX. These compounds have shown limited efficacy and severe side effects, highlighting the necessity for earlier detection and the development of more effective, and better-tolerated treatments. Although targeted therapies are promising for the treatment of several types of cancer, identification of suitable targets for early diagnosis and targeted therapy of PDAC is challenging. Interestingly, several transmembrane proteins are overexpressed in PDAC cells that show low expression in healthy pancreas and may therefore serve as potential targets for treatment and/or diagnostic purposes. In this review we describe the 11 most promising transmembrane proteins, carefully selected after a thorough literature search. Favorable features and potential applications of each target, as well as the results of the preclinical and clinical studies conducted in the past ten years, are discussed in detail.

## Introduction

Pancreatic ductal adenocarcinoma (PDAC) is the fourth cause of cancer-related deaths in the world 1. Although several oncogenic mutations such as *KRAS*, *CDKN2 A*, *TP53*, *SMAD4*, *GNAS*, and *RNF43* have been identified, the pathophysiology and genetic profile of PDAC are complex and heterogeneous 1,2. In addition to gene mutations, overexpression of several transmembrane proteins such as growth factor receptors, mucins, tight junction and adhesion proteins, which are crucial for the activation of oncogenic pathways, have been reported in PDAC 2-4. Moreover, the dense stroma and extracellular matrix surrounding the tumor cells create a hypovascular and hypoxic microenvironment that not only supports proliferation and immune evasion but also interferes with drug delivery, adding to the complexity of this disease 1.

The incidence of PDAC is increasing, while current treatments have not significantly improved the life expectancy over the last few decades, with less than 10% of PDAC patients reaching a 5-year overall survival 5. This high mortality rate is mostly due to the fact that up to 80% of PDAC cases are diagnosed in advanced stages of the disease, limiting the treatment options 6. Early-stage symptoms such as indigestion, abdominal pain and nausea, are generally unspecific and, in some cases, patients are asymptomatic until the later stages, allowing the disease to escalate unnoticed. Moreover, even after surgical resection, PDAC frequently recurs and shows limited response to current chemotherapeutic agents 6. Therefore, there is a tremendous need for more effective diagnostic and therapeutic options.

A considerable number of preclinical studies have investigated potential novel therapeutic options for PDAC, but only few reached the clinic, showing limited efficacy and an increase in survival by only a few months 6. Currently approved treatment options include surgery, chemotherapy and more recently targeted therapies, namely tyrosine kinase inhibitors such as erlotinib. In general, targeted therapies are meant to block specific target molecules involved in the growth and progression of cancer 7,8. Surgery in combination with adjuvant therapy, such as chemoradiotherapy, is usually the recommended option for resectable PDAC. In spite of the initial effects seen after treatment, relapse is very common 9. Moreover, up to 85% of PDAC cases are non-resectable due to presence of distant metastases and/or locally advanced disease 9,10. For these patients, chemotherapy with gemcitabine or FOLFIRINOX is usually recommended as single agent or in combination therapies. However, these agents have shown limited efficacy and often come with severe side effects that have a substantial impact on patients' quality of life.

Many studies focused on the identification and targeting of PDAC-specific proteins that can potentially serve as targets for novel therapies with higher efficacy and fewer adverse effects 11. A variety of targeted therapies, namely monoclonal antibodies and antibody-drug conjugates are currently under clinical investigation for treatment of PDAC. These therapies are usually administered in combination with gemcitabine. For instance, erlotinib, a tyrosine kinase inhibitor, in combination with gemcitabine has been shown to increase survival compared to gemcitabine alone 11,12. Alternatively, immunotherapy has also been utilized for the treatment of PDAC, as recently reviewed 13. Different types of immunotherapy have been investigated, such as the use of patients' own chimeric antigen receptor (CAR) T cells directed against cancer cells, cancer vaccines, monoclonal antibodies specifically targeting immune components, and conjugates of these with drugs (also known as antibody-drug conjugates) 4,6.

Antibody-targeted photodynamic therapy (PDT), known as photoimmunotherapy (PIT), has also been investigated preclinically for the treatment of PDAC 14-16. This approach makes use of photosensitizers (PS) that are specifically delivered to the tumor tissue by means of monoclonal antibodies or other targeting moieties. Activation of PS at the tumor site after illumination results in reactive oxygen species production which subsequently induces tumor cell death 14,15. While targeted PDT and PIT strategies have not yet reached clinical trials in PDAC, preclinical studies have so far shown promising results, highlighting the potential of this therapeutic strategy 15,16.

Another type of targeted therapy is receptor-targeted radionuclide therapy (RTRT), in which radionuclides conjugated to peptides, antibodies or antibody fragments deliver radiation specifically into the tumor 17. RTRT has shown to achieve significant improvements both in diagnostics and therapeutic efficacy in several clinical studies, especially in patients with neuroendocrine tumors and prostate cancer 17-19. By using different radionuclides for diagnostics and therapeutics, the attractive “theranostic” approach can be used for patient identification, selection and treatment. Nonetheless, few clinical studies have investigated such approaches for treatment of PDAC, with variations both in inclusion criteria and results, indicating that additional studies are required to further explore the full potential of this approach 17.

Based on an extensive literature review and specific criteria used for selection, we propose the 11 most promising proteins that may serve as targets for PDAC diagnostics and/or therapies. In particular, these targets can be explored for delivery of cytotoxic compounds, namely radionuclides and drugs, conjugated to targeting moieties that specifically associate with these proteins on tumor cells. This information can guide further preclinical studies and may contribute to the management of patients with PDAC in the future.

## Search strategy and target selection

**Figure 1:**
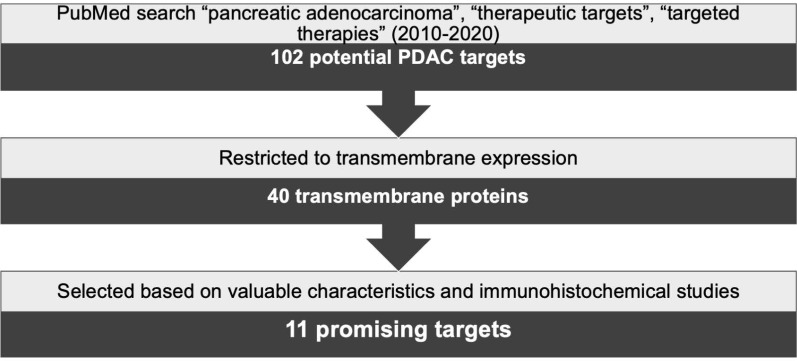
Schematic representation of target selection for the diagnosis and treatment of PDAC.

A thorough search was conducted using PubMed and combinations of the keywords “pancreatic adenocarcinoma”, “therapeutic targets” and “targeted therapies”. Results were limited to reviews published between 2010 and 2020 yielding 102 possible target proteins. Next, this number was reduced by selecting the targets showing transmembrane expression, resulting in 40 proteins. In the subsequent steps, a literature research was implemented to establish, for each target, the frequency and level of expression in PDAC, its expression in healthy tissues, and the results from preclinical *in vivo* or clinical studies. In addition, immunohistochemical expression of these targets in PDAC was assessed based on published studies in consultation with an experienced pathologist (LAAB). Protein expression was verified to be solely or mostly confined to cancer cells and not to the stroma, while also being present in the majority of the cancer cells. Based on this strategy, schematically represented in Figure 1, 11 targets were selected as the most promising targets to be explored for PDAC diagnosis and/or therapies. The characteristics of these selected transmembrane proteins are summarized in Table 1.

**Figure 2:**
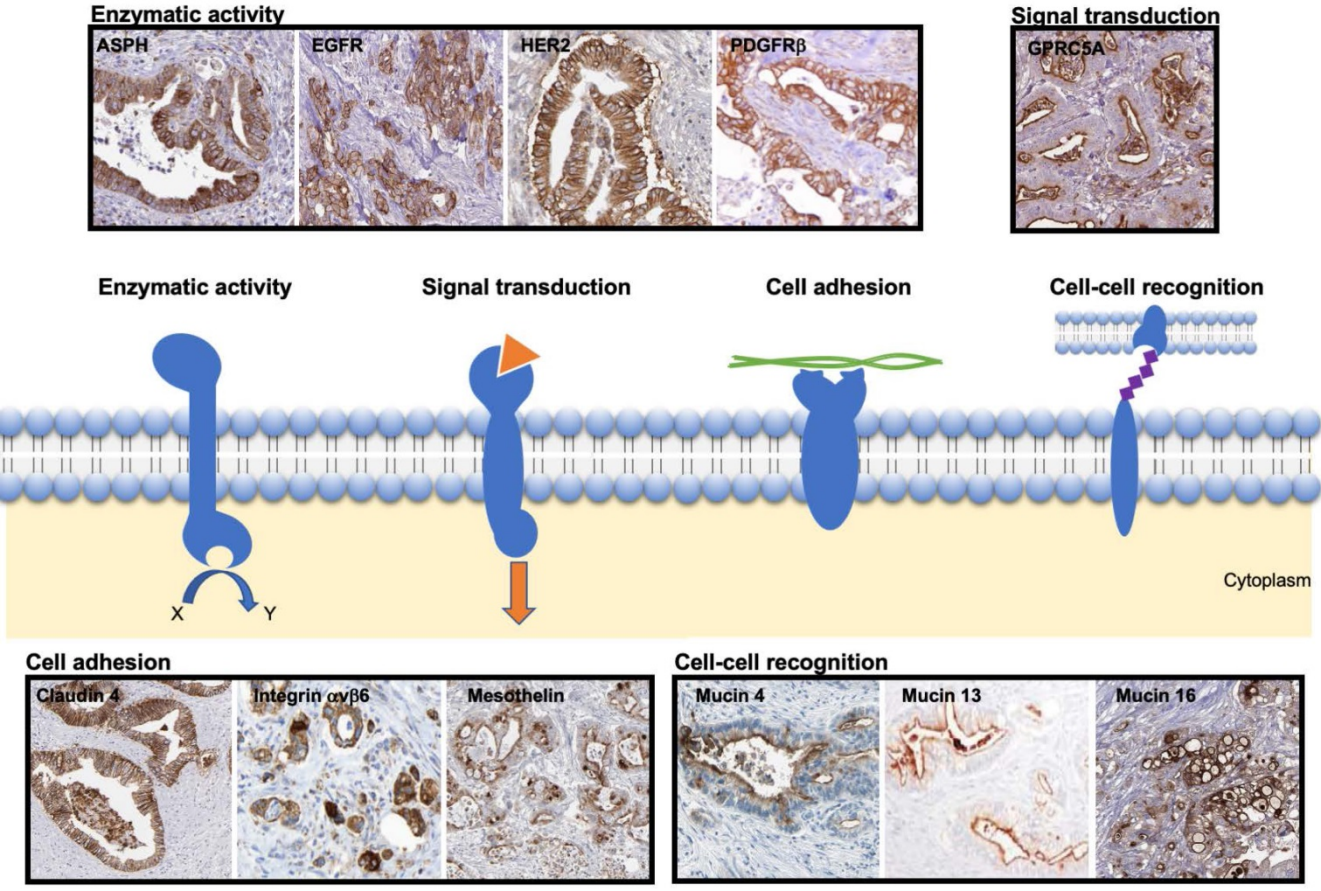
Representative immunohistochemical staining pattern of the selected transmembrane proteins in patient-derived PDAC samples. The scheme presents transmembrane proteins grouped based on their function, i.e. enzymatic activity, signal transduction, cell adhesion or cell-cell recognition. The images were adapted from peer-reviewed papers or Protein Atlas (www.proteinatlas.org/). Enzymatic activity: ASPH 50 EGFR 51, HER2 52, PDGFRβ 25 (copyright 2016 Springer Nature); Signal transduction: GPRC5A 53; Cell adhesion: Claudin 4 54, Integrin αvβ6 55 (copyright 2019 John Wiley and Sons), Mesothelin 56; Cell-cell recognition: Mucin 4 57, Mucin 13 58 (copyright 2015 Spandidos Publications), Mucin 16 59. ASPH: ASPH: Aspartate-β-hydroxylase; EGFR: epidermal growth factor receptor; GPRC5A: G protein-coupled receptor family member 5 group A; HER2; human epidermal growth factor receptor 2; PDGFRβ: platelet derived growth factor receptor beta.

Based on the described strategy, the most promising transmembrane proteins can be grouped into four categories based on their function, as illustrated in Figure 2:

- Enzymatic activity: ASPH, EGFR, HER2 and PDGFRβ;
- Signal transduction: GPRC5A;
- Cell adhesion: Claudin 4, Integrin αvβ6, Mesothelin;
- Cell-cell recognition: Mucins (4, 13 and 16).

In the next sections, for each of these transmembrane proteins, details about their expression in cancer and normal tissue are provided. Representative immunochemistry staining patterns of these proteins are shown in Figure 2. In addition, encouraging preclinical studies, and, when available, clinical studies are described in the following sections. An overview of the transmembrane proteins described in this review is summarized in Table 2.

### Transmembrane proteins with enzymatic activity

#### ASPH

Aspartate-β-hydroxylase (ASPH) is a type II transmembrane protein which belongs to the α-ketoglutarate-dependent dioxygenase family. This receptor consists of a cytoplasmic domain, a transmembrane domain, a luminal region, and a C-terminal catalytic domain. ASPH has an important role in embryonic development where it promotes organ formation and growth but shows virtually no expression in healthy adult tissues, mostly limited to the placenta 9.

Expression of ASPH is aberrantly increased in PDAC and also other cancers such as hepatocellular carcinoma and prostate cancer, and has been reported to contribute to proliferation, migration, invasion, recurrence, and tumor invasiveness 9,89,90. Although the exact tumor-promoting mechanisms of ASPH have not been elucidated yet, it has been hypothesized that this protein might be involved in several tumorigenic pathways, including the Notch pathway. This will subsequently result in PDAC progression, somatic mutations of mitochondrial DNA, as well as inhibition of natural killer cells function, thus protecting tumor cells from immune surveillance 9. To our knowledge, expression of ASPH in the tumor microenvironment has not been reported so far.

In two independent studies, ASPH upregulation has been reported in 97% of 101 10 and of 104 60 patient-derived PDAC samples, while its expression was not detected in normal pancreas. Moreover, the upregulation of this receptor has been shown in precursor lesions and increases during neoplastic progression, indicating that ASPH could be a promising diagnostic and therapeutic target for PDAC from the early stages of the disease 60. So far, no ASPH-targeted agents have been assessed in clinical trials and only few have shown promising results in preclinical studies 91,92. In a PDAC patient-derived xenograft murine model, the ASPH-targeting antibody-drug conjugate SNS-622-DM1 inhibited tumor growth, likely by inducing cell cycle arrest and promoting apoptosis. In the same model, SNS-622-DM1 could also inhibit lung metastasis while being well-tolerated 10. In another study, the efficacy of MO-I-1182, a small molecule targeting the enzymatic activity of ASPH, was investigated in a patient-derived murine xenograft model for PDAC in both primary tumors and metastases. MO-I-1182 effectively and specifically targeted ASPH activity, thereby inhibiting PDAC growth, progression and suppression of metastases to the lungs 60.

#### EGFR

The epidermal growth factor receptor (EGFR) is a transmembrane glycoprotein which belongs to the ErbB family of receptor tyrosine kinases. This receptor contains an extracellular-binding domain, a transmembrane region and a cytoplasmic region 4. EGFR is activated through binding of a variety of growth factors, such as epidermal growth factor, transforming growth factor-α, amphiregulin, and epigen. Upon ligand binding, EGFR undergoes conformational changes that lead to the dimerization and phosphorylation of the cytoplasmic domain, which induces a signaling cascade of intracellular proteins that ultimately contribute to cell survival, growth, differentiation, and proliferation in healthy tissues 61.

EGFR overexpression has been reported in various types of cancer, including in up to 90% of PDAC cases. Overexpression has been reported in late stage precursor lesions and PDAC, and it is significantly increased in tumor tissues compared to healthy pancreatic tissue 4,62. In PDAC, EGFR is involved in the activation of pathways that contribute to tumor proliferation, metastasis, malignant transformation, survival, and drug resistance 11,20. RAS/RAF/MEK/ERK and RAS/PI3K/PTEN/Akt/mTOR are the most common activated pathways in PDAC, not only due to the EGFR overexpression, but also as a result of *KRAS* mutation 4,20. Although *KRAS* mutation was shown to inhibit the efficacy of the EGFR-targeted therapies in non-small cell lung cancer, controversial results were obtained in PDAC. Therefore, further research is required to confirm the effect of *KRAS* in EGFR targeted therapies 93,94. In addition, EGFR is expressed on endothelial cells of the tumor microenvironment, where it contributes to angiogenesis, proliferation and metastasis through stimulation of vascular endothelial and fibroblast growth factors, and interleukin-8 4,63.

Because of the long-standing history of research on EGFR and its important role in PDAC, this receptor has been investigated extensively for targeted therapies. Most studied agents are the anti-EGFR targeted monoclonal antibody cetuximab and kinase inhibitors such as erlotinib 4,11,12. However, these treatments failed to improve PDAC therapy substantially. Therefore, several other EGFR targeted agents that have shown promising results in preclinical and early clinical stages, and particularly in combination therapies, are currently under investigation 4,95,96. For example, Li and colleagues explored the anti-EGFR monoclonal antibody RC68 conjugated to the cytotoxin monomethyl auristatin E (MMAE) in a murine xenograft model. In this study, the RC68-MMAE conjugate successfully inhibited tumor growth and even induced tumor regression, while being well tolerated 96. Recently, EGFR was also targeted for photodynamic therapy using cetuximab photoimmuno-nanoconjugates *in vivo* 16. The nanoconjugates were shown to have high efficacy and good tolerability, providing a promising strategy for the treatment of PDAC. EGFR-targeted agents have also been investigated in combination with radiotherapy due to the role of EGFR overexpression in radiotherapy resistance. Combinations of cetuximab and radiotherapy have shown promising results both in terms of efficacy and tolerability in preclinical and early stages of clinical studies 4,95. The combination of EGFR-targeted therapies with cytotoxic agents is also extensively investigated, mostly with gemcitabine, both agents in combination with tyrosine kinase inhibitors (NCT00075686, NCT00040183), or the use of antibody-drug conjugates to achieve selective and specific delivery of cytotoxic agents to the tumor cells 4,20. An overview of the approved clinical trials targeting EGFR is summarized in Table 3.

While the therapeutic potential of EGFR-targeted therapies is thoroughly studied, it is also under investigation for imaging purposes. For instance, EGFR and vascular endothelial growth factor were simultaneously targeted for fluorescence imaging using a bispecific fusion protein in an orthotopic PDAC murine model, showing promising results 97. Another study using monoclonal antibody fragments for microPET/CT imaging was also able to visualize pancreatic tumors in a xenograft mouse model 98.

#### HER2

HER2 is a member of the ErbB family of receptor tyrosine kinases which consists of four domains in the extracellular region, a single membrane-spanning domain and an intracellular region comprising a juxtamembrane domain, a tyrosine kinase domain, and a C-terminal tail. Although HER2 has no natural ligand, this protein is involved in several cell growth signaling pathways through heterodimerization with other ErbB receptors 22.

While the exact role of HER2 in PDAC has not yet been confirmed, activation of this receptor has been correlated with the cell proliferation, migration and survival 23. HER2 is thought to exert its activity through activation of several tumorigenic intracellular pathways, such as PI3K/Akt/mTOR and RAS/RAF/MEK/ERK1/2 to enhance tumor cell growth, proliferation and invasion 22. Moreover, HER2 was shown to interact with other proteins which are overexpressed in PDAC, including EGFR, MUC4 and MUC13. These interactions can result in the activation of the tumorigenic pathways MAPK, JNK and STAT, leading to the tumor proliferation and metastasis 42. Several studies have reported a correlation between the expression of HER2 with the development of metastasis and poor survival, as well as tumor differentiation 61,99,100. To our knowledge, the expression and role of HER2 in the PDAC microenvironment has not been explicitly reported.

Low levels of HER2 expression are present in various normal tissues and not healthy pancreas 52,101. In contrast, overexpression of this receptor has been observed on the cell membranes of 0-82% of PDAC samples. Nonetheless, the correlation of clinicopathological features of PDAC showing significant HER2 expression is unclear, and contradictory results have been reported in literature 65.

In addition to PDAC, HER2 is one of the main receptors overexpressed in breast cancer, making it a well-characterized target. Several therapeutic agents such as monoclonal antibodies trastuzumab and zenocutuzumab, have been developed and tested in clinical trials for PDAC treatment. Moreover, the efficacy of tyrosine kinase inhibitors has also been assessed in the clinical studies for several types of cancer, including PDAC, showing contradictory results with a number of studies reporting little to no efficacy, while others indicated more favorable outcomes. For instance, trastuzumab has not yet displayed improvement over chemotherapy in clinical studies 11,23. On the other hand, HER2-specific CAR T cell therapy in a phase I trial has shown promising results 13. Cancer vaccines such as ETBX-021 are also assessed in clinical trials, though results for PDAC have not yet been published (NCT03586869). An overview of the clinical trials targeting HER2 is summarized in Table 3.

#### PDGFRβ

Platelet-derived growth factor receptor beta (PDGFRβ) is one of the two isoforms of PDGFR and, like EGFR and HER2, it belongs to the receptor tyrosine kinase family. The structure of PDGFRβ presents an extracellular part consisting of five immunoglobulin-like domains, a transmembrane domain, and, intracellularly, a juxtamembrane domain, a tyrosine kinase domain, and a C-terminus 102. Like other receptor tyrosine kinases, PDGFRβ requires dimerization to be activated upon ligand binding. This is achieved either through homo- or heterodimerization, the latter of which can involve other receptors, such as EGFR or integrins, leading to the activation of various signaling pathways 102. Under healthy conditions, PDGFRβ is mainly involved in blood vessel formation during embryonic development, while in adults it displays roles in wound healing and the control of interstitial fluid pressure 102.

In PDAC, PDGFRβ levels were detected on the cell membrane of most or all of the patient-derived samples, with nearly 50% showing particularly high expression 25,70. On the other hand, expression in healthy pancreatic tissue was not detected, making this receptor suitable as a potential target in PDAC 70,71. The potential of this target is further supported by its role in disease progression, as studies have indicated that PDGFRβ contributes to several tumorigenic processes and is particularly involved in cell invasion and metastasis. In addition, PDGFRβ is consistently expressed in the tumor microenvironment, where it promotes angiogenesis via the tumor stroma, further contributing to the tumor progression and metastasis 26. The metastasis-promoting activity of PDGFRβ might also be related to mutant p53. In fact, mutant p53 is thought to induce PDGFRβ expression, enhancing metastatic potential and thus correlating with poor prognosis 25,26. In fact, the angiogenic effects of PDGFRβ were shown to negatively affect radiation treatment efficacy, and it is suggested that this receptor could influence transport of therapeutic agents to the tumor site by regulating interstitial fluid pressure 103.

Until now, PDGFRβ-targeted therapies that have reached the stage of clinical trials are mainly small molecule tyrosine kinase inhibitors (Table 3). These inhibitors have shown promising results in preclinical studies, where therapeutic agents such as masitinib and sunitinib could suppress tumor growth and increase PDAC sensitivity to radiation 104,105. Although clinical trials on neuroendocrine pancreatic tumors showed positive results, the effects on PDAC were not encouraging 106. The reason for these discrepancies has not been fully elucidated but might be partially caused by interstitial fluid pressure. Ongoing clinical trials might provide further insights on the effectiveness of tyrosine kinase inhibitors (NCT03766295, NCT02349867). To our knowledge, no other PDGFRβ-targeted therapies are currently under investigation in PDAC than tyrosine kinase inhibitors.

### Signal transduction transmembrane protein

#### GPRC5A

G protein-coupled receptor family C member 5, group A (GPRC5A), also known as retinoic acid-inducible 3 (RAI3), is a transmembrane protein which is part of the large protein superfamily of G protein coupled receptors 72. The structure of GPRC5A is characterized by seven membrane-spanning domains connected by three extracellular and three intracellular loops, with an intracellular C-terminus and a short extracellular N-terminus 29. The exact role of GPRC5A, both in cancer and normal conditions, has not been fully elucidated, though it is thought to be related to cell cycle progression and involvement of this protein in several signaling pathways 30. In particular, GPRC5A is involved in the activation of proteins and signaling pathways often related to tumor development, such as NF-κB, STAT, and FAK/Src. However, studies have reported both tumor-promoting and tumor-suppressing activity of GPRC5A in these pathways, indicating that the role of this receptor is possibly tumor dependent 29.

In healthy conditions, GPRC5A is predominantly expressed in lung tissue, with minor expression in intestines, kidneys, thyroid, bladder, and low to non-detectable expression in pancreas 28-30. However, in PDAC the expression of GPRC5A is significantly increased, showing strong membranous expression in up to 82% of 376 patient-derived samples 28. GPRC5A expression in PDAC appears to be mostly limited to the cancer cells and not present in the surrounding microenvironment 107. Of note, while GPRC5A expression was shown to be consistently upregulated in certain types of cancer, including PDAC, downregulation was observed in others, such as non-small cell lung cancer and hepatocellular carcinoma 29. In PDAC, GPRC5A is involved in tumor proliferation, invasion and metastasis 28,29. The potential comprehensive roles of this protein in several aspects of cancer development, plus its frequent overexpression in PDAC, make GPRC5A a potentially promising therapeutic and diagnostic target. In addition, GPRC5A also plays a role in the development of chemoresistance, thus making this protein a potential target to increase the efficacy of the chemotherapeutic agents 28,29. To our knowledge, so far no therapeutic or diagnostic agents targeting GPRC5A have been investigated in clinical trials. This could be due to the lack of detailed insight on the role of this protein in PDAC, indicating further studies are needed to explore the potential of this target in PDAC for therapeutic and diagnostic applications.

### Cell adhesion transmembrane proteins

#### Claudin 4

Claudin 4 is one of the 27 members of the claudin family of transmembrane proteins, composed of four transmembrane domains and two extracellular loops. Claudins have functional and structural activity in the composition of the tight junction barrier in normal epithelium 3,73,74. As part of the tight junction barrier, claudins have several roles in normal cell functioning, including the maintenance of cell polarity and adhesion, as well as controlling of traffic of substances between cells 31. Several studies have shown that claudin expression is dysregulated in various types of cancer, where it contributes to tumor progression and invasion 3,73. This is likely due to the loss of cell adhesion and polarity caused by tight junction barrier disruption, which is one of the hallmarks of tumorigenesis 75,108.

Overexpression of claudin 4 has been reported in a variety of cancers, including PDAC, with high and diffuse expression detected in 70-97% of patient samples 73,74. Claudin 4 expression was shown to be restricted to the tumor cells and not present in the surrounding stroma 109. Importantly, overexpression of claudin 4 in PDAC has been found not only in primary tumors and metastases but also in precursor lesions, making it a potential target for early diagnosis and treatment 75. Moreover, the role of claudin 4 in the development of chemoresistance by inhibiting access of therapeutic agents to tumor cells has been reported in several studies 31-33. Therefore, agents able to interfere with the function of this protein may improve therapeutic efficacy of chemotherapeutic regimens, reduce the side effects, and improve patients' quality of life.

Two main approaches have been described for targeting of claudin 4. One strategy makes use of *Clostridium perfringens* enterotoxin (CPE). The C-terminal fragment of CPE (C-CPE) is one of the ligands of claudin 4, and therefore C-CPE-fused proteins and CPE could be utilized to target claudin 4. However, this strategy has revealed some drawbacks, particularly the immunogenicity of CPE and C-CPE-fused proteins, and poor selectivity due to the possible interaction of CPE with other members of the claudin family 108. The second approach relies on the use of monoclonal antibodies to target the extracellular domains of claudin 4. However, development of anti-claudin targeted antibodies has shown to be challenging. This is mainly due to the fact that claudins have small extracellular domains (~18-50 amino acids) and high inter-species similarity, causing low immunogenicity. Nonetheless, a number of monoclonal antibodies against human claudin 4 have been developed that show both anti-tumor effects and induction of chemosensitivity *in vivo* 31,108. Sasaki and co-workers investigated the effects of anti-claudin 4 antibody 4D3 in combination with FOLFIRINOX in a PDAC subcutaneous murine tumor model. The combination of the 4D3 antibody and FOLFIRINOX showed significant anti-tumor effects while the adverse events were significantly reduced due to the lower FOLFIRINOX dose. The increased activity seen for co-administered agents has suggested the induction of chemosensitivity by anti-claudin 4 agents in addition to its anti-tumor effects 31.

As an early-stage marker, claudin 4 has also been investigated for imaging and diagnosis. Several studies have explored application of CPE and monoclonal antibodies for the imaging of PDAC *in vivo*, showing promising results 75,110,111. To our knowledge, claudin 4 has not been investigated yet in any clinical trials for the treatment or diagnosis of pancreatic cancer 91,92.

#### Integrin αvβ6

Integrin αvβ6 is a heterodimeric cell surface receptor with two subunits, α and β, both being a type I transmembrane glycoprotein. Each subunit comprises a short cytoplasmic domain, a transmembrane domain, and a large extracellular region projecting from the membrane 112. Integrins are mainly involved in cell adhesion, cell-extracellular matrix interactions, as well as intracellular and cell-cell signaling 36,112,113.

Aberrant expression of several integrins has been implicated in various cancers, with both inhibitory and stimulatory functions in tumor proliferation, metastasis and invasion 35. Dysregulation of integrin αvβ6 has been shown in numerous cancers, including PDAC, where its expression is progressively upregulated during neoplastic progression from precursor lesions to invasive adenocarcinoma and metastasis. Several studies have reported integrin αvβ6 upregulation, with diffuse membranous and cytoplasmic expression in nearly 90% of PDAC samples, whereas healthy pancreas and other healthy epithelia show low or undetectable expression 35,77,78. Expression of this protein was reported to be mostly restricted to the tumor cells, though a few samples showed limited expression in the surrounding stroma and blood vessels 36.

The role of integrin αvβ6 in PDAC has not yet been completely elucidated, although it is partly involved in activation of transforming growth factor-β (TGF-β) 36,113. The growth suppressing effect of TGF-β has negligible effect on tumor cells, while healthy cells are greatly affected. This subsequently results in growth inhibition and proliferation of healthy cells at the tumor site, thereby facilitating tumor proliferation invasive growth 113. Moreover, increased levels of TGF-β possibly contribute to other tumor-promoting processes, such as epithelial-mesenchymal transition, metastasis, and immune evasion 113.

Despite the significance of integrin αvβ6 in PDAC, therapeutic agents targeting this protein have not yet reached clinical trials. However, a number of preclinical studies have shown promising results. Recently, the anti-integrin αvβ6-targeted antibody 264RAD was tested in combination with gemcitabine in a human xenograft model of PDAC in mice 55. In this study, combining 246RAD with gemcitabine showed significant tumor growth reduction compared to control and monotherapy, with three subjects achieving full recovery. These effects were likely the result of reduced tumor cell proliferation, growth signaling, and also increased apoptosis. Another study investigated the potential of integrin αvβ6-targeted for photodynamic therapy *in vivo* 15. The study assessed the targeted near-infrared agent Dye-SA-B-HK (IRDye700-Streptavidin-biotin-HK), in a subcutaneous mouse model of PDAC. The results showed that the treatment was well-tolerated and had significant anti-tumor effects. The authors proposed targeted PDT as a potential strategy for the treatment of PDAC, although clinical studies are required to substantiate these findings.

As opposed to the therapeutic agents, imaging strategies targeting this integrin have been more thoroughly investigated and some are currently in clinical trials. Several of these studies include the integrin αvβ6-targeting radiotracers ^18^F-αvβ6-binding-peptide (NCT03164486) and [^18^F]-R01-MG-F2 (NCT02683824); although, results have not yet been published. An overview of the clinical trials targeting αvβ6 is given in Table 3.

#### Mesothelin

Mesothelin is a glycoprotein normally found on the surface of mesothelial cells in the pleura, pericardium and peritoneum 81,82. It is a glycosylphosphatidylinositol-anchored membrane protein formed from a precursor which is further processed into the soluble serum mesothelin relative peptide (SMRP) 37,80. The role of mesothelin in healthy cells has not been identified yet, although it has been suggested that mesothelin might have a role in cell adhesion 38,114. Preclinical studies have shown that this protein is not essential for normal functioning and development 38,114. In PDAC, expression of mesothelin was reported in up to 100 of patient-derived samples and overexpression was found in 80-85% cases, while no expression was observed in healthy pancreatic tissue 37,80. Expression of mesothelin has been shown to be limited to the tumor cells, which could be clearly distinguished from the mesothelin-negative surrounding stroma 80.

In several types of cancer, aberrant expression of mesothelin has been shown to play a role in angiogenesis, apoptosis resistance as well as promoting tumor proliferation, invasiveness and metastasis 37. The mechanisms by which mesothelin exerts its tumorigenic effects have been thoroughly investigated 37. Mesothelin is involved in a variety of tumor-promoting pathways such as STAT3, NFκB, ERK, and PI3K/Akt. Moreover, it has been shown to influence the expression of PDAC-related proteins, such as BAX and BCL-2. Activation of Akt/PI3K/NFκB and the MEK1/2 pathways lead to apoptosis resistance, which further contributes to the tumor survival 37. In addition, mesothelin plays a role in metastasis through binding to MUC16, leading to enhanced matrix metalloproteinase-7 activity thereby promoting angiogenesis in metastatic tissues 37. Finally, mesothelin is involved in epithelial-mesenchymal transition, a process known to contribute to tumor invasiveness 37. A correlation between mesothelin and chemoresistance has also been proposed, but the underlying mechanisms are unclear 37.

Given its involvement in tumorigenic processes and its marginal expression in healthy tissue, mesothelin has been extensively investigated as a therapeutic target for cancer. Currently, the main focus for mesothelin-targeted therapies is the use of anti-mesothelin antibodies for the development of immunotoxins and antibody-drug conjugates. Some of these treatments have shown promising results *in vivo* and have advanced to early phase clinical trials 37,91,115. The antibody-drug conjugate anetumab ravtansine was tested in 148 adult patients with advanced, metastatic or recurrent solid tumors in a phase I clinical study. Anetumab ravtansine is composed of an anti-mesothelin monoclonal antibody conjugated to the tubulin inhibitor DM4. The phase I study showed an adequate safety profile with manageable side effects, and preliminary data indicated that anetumab ravtansine showed promising anti-tumor effects in mesothelin-expressing tumors 116. Monoclonal antibodies against mesothelin have also been radiolabeled with thorium-227 (BAY2287411) for radioimmunotherapy. BAY2287411 exhibited anti-tumor effects *in vivo* and is currently studied for PDAC and other solid tumors in a phase I clinical trial (NCT03507452) 37. CAR-T cell therapy and vaccines directed against mesothelin have also shown promising results in preclinical studies and are presently undergoing clinical trials (e.g. NCT03497819, NCT03122106) 37,91,115.

In addition to therapy, mesothelin is being investigated as a target for imaging of PDAC, although studies have not reached clinical trials yet. A number of imaging strategies have been tested *in vivo*, including a technetium-99m labeled anti-mesothelin antibody for SPECT-CT imaging and a nanoprobe modified with an anti-mesothelin antibody 117,118. Results have so far been promising, suggesting that mesothelin-targeted agents could be suitable tools for visualization of PDAC. An overview of the clinical trials targeting mesothelin is summarized in Table 3.

### Cell-cell recognition transmembrane proteins

#### Mucins

Mucins are a family of 21 high-molecular-weight glycoproteins expressed on different types of epithelial tissues, whose functions vary from protecting mucous membrane barriers to contributing to cellular regeneration, differentiation, adhesion and signaling 40,41. Mucins comprise membrane-bound MUC1, MUC3, MUC4, MUC11-13, MUC15-17, MUC20-21 and, secreted MUC2, MUC5AC, MUC5B, MUC6, MUC7, MUC19. The focus of this review is solely on the membrane-bound mucins. The structure of mucins is characterized by the presence of tandem repeat regions, to which several glycans are linked. In addition, these proteins contain various functional domains and a transmembrane domain is also found in membrane-bound mucins 40,41.

Aberrant expression of several mucins has been reported in pancreatic cancer, increasing steadily through neoplastic progression 40. These proteins are thought to have numerous functions in the pathophysiology of PDAC. In fact, dysregulation of mucin expression can lead to changes in the mucin mesh found on the cell surface, which might affect drug absorption and subsequently cause chemoresistance and immune evasion 40,41. In addition, aberrant expression of certain mucins possibly leads to enhanced pro-tumorigenic signaling, and also their role in cell adhesion contributes to tumor invasion and metastasis 41. Due to the important role of mucins from the very early stages of PDAC, several members are considered as potential diagnostic and therapeutic targets. The membrane bound mucins, MUC4, MUC13 and MUC16 have shown most promising properties and therefore are discussed in more detail.

#### MUC4

MUC4 is one of the most frequently overexpressed mucins in PDAC, and in recent years several studies have been focusing on its potential as a therapeutic target. MUC4 is a glycoprotein composed of two subunits, α and β. The extracellular domain forms subunit MUC4α, which contains a tandem repeat region of variable length, a nidogen-like and an adhesion associated-domain in MUC4 and other proteins functional domains. Whereas subunit MUC4β comprises a cytoplasmic tail, a transmembrane domain and, extracellularly, one Von Willebrand factor type D domain, and three EGF-like domains 42.

MUC4 expression in healthy tissues is mainly seen in epithelial cells of the airways and in secretory fluids such as saliva, breast milk and tears 42,84. While MUC4 in normal pancreatic tissue has been consistently reported to be undetectable, expression has been observed in precursor lesions and increases during neoplastic progression, with positive expression in 70 to nearly 95% of PDAC cases 85,86.

The role of MUC4 in PDAC has been shown to be fairly extensive, with involvement of this mucin with several other proteins, promoting tumorigenic processes. One of the main interactions is with growth factor receptors, particularly HER2 and HER3, via the EGF-like domains on the extracellular domain of MUC4. With this interaction, MUC4 mediates HER2 and HER3 activation, thus enhancing signaling cascades such as MAPK, JNK, STAT-1, promoting tumor proliferation and metastasis 40-42. MUC4 was also recognized to play a role in E- and N-cadherin signaling, further enhancing proliferation and contributing to epithelial-mesenchymal transition 42. Moreover, studies have indicated MUC4's contribution to immune evasion by numerous mechanisms, such as epitope masking and apoptosis of MUC4 specific T-cells. Finally, MUC4 appears to play a role in gemcitabine chemoresistance, by inhibiting gemcitabine-induced apoptosis and increasing the level of transporters that remove gemcitabine from the tumor cells 42. MUC4 is also known to be involved in the interaction between the tumor cells and surrounding environment 42. However, to our knowledge studies have not focused on detecting its expression in the tumor microenvironment.

With such an important role in PDAC, MUC4 is being evaluated as a potential therapeutic target, especially in the field of immunotherapy. In fact, like other cancer-associated mucins, MUC4 not only is overexpressed in PDAC but also displays aberrant glycosylation patterns which is absent in healthy tissues, making it a potential target for cancer vaccines 42,119. While no MUC4-targeted vaccines have been studied in clinical trials, a few have been evaluated *in vivo*, with promising results 120,121. Recently, Liu *et al.* developed a MUC4 nanovaccine by encapsulating a recombinant MUC4β subunit in nanoparticles. To assess antibody response, they immunized mice using the nanovaccine and analyzed the serum, showing the presence of MUC4β-specific antibodies, which were not detected in the control group 120. The potent immune response induced by this and the other MUC4 vaccines *in vivo* suggests that this protein could have a significant role as a target for immunotherapy in PDAC.

In addition to the therapeutic potential, the frequent overexpression and aberrant glycosylation of MUC4 makes it a potential target for imaging. To this end, a recent study investigated both the therapeutic and imaging potential of MUC4 and two other targets using single-chain antibodies conjugated to magnetic iron oxide nanoparticles, in a subcutaneous mouse model of PDAC. The conjugate successfully acted as an MRI negative contrast agent and also showed anti-tumor effects *in vivo* 121.

#### MUC13

MUC13 is less known compared to the other mucin family members, but the studies published reveal this protein as a potential target in PDAC. MUC13 presents a short transmembrane domain, a cytoplasmic domain and a large extracellular portion composed of a tandem repeat domain, three EGF-like domains and a sea urchin sperm protein, enterokinase and agrin (SEA) domain 43.

MUC13 is normally expressed in the healthy trachea, stomach, intestines and kidneys, while expression in normal pancreas is virtually absent 43,45. However, MUC13 expression is highly upregulated in PDAC. In fact, immunohistochemical studies of over 200 patient-derived samples confirmed membrane, cytoplasmic and nuclear localization of MUC13 in nearly 95% of the PDAC cases. Moreover, MUC13 expression was detected in all 29 precursor lesions, with increasing levels following the disease progression 45. The expression of MUC13 in PDAC tumor microenvironment has not been reported yet.

Similar to MUC4, MUC13 has been shown to interact with HER2, likely through binding of HER2 with the EGF-like functional domains of MUC13. *In vivo* studies have reported elevated expression levels of HER2 in MUC13 overexpressing tumors, indicating that this mucin might affect the expression and activation of HER2 43,44. Activation of HER2 would subsequently lead to downstream signaling of oncogenic pathways PI3K/Akt and MAPK, as described in the HER2 section of this review. In addition, MUC13 is suspected to play a role in the expression of other oncogenic proteins, such as PAK1 and S100A4 which play role in tumor migration, invasion and metastasis. Furthermore, MUC13 overexpression was correlated with an increase in lymph node metastasis *in vivo*, supporting that MUC13 likely plays a role in these processes 43-45. In addition to its role in the activation of pro-oncogenic proteins, MUC13 overexpression has been linked to downregulation of the tumor suppressing protein p53 43. Overall, these findings point to a significant role of MUC13 in PDAC tumorigenesis 43-45.

With such a frequent overexpression of MUC13 and an extensive role in the pathophysiology of PDAC, this protein is a potentially interesting target for both diagnostic and therapeutic purposes. Nonetheless, MUC13 has not been gaining as much attention as other members of this family and, to our knowledge, no MUC13 targeting-based treatments have been tested in preclinical or clinical studies.

#### MUC16

MUC16, also known as carbohydrate antigen (CA) 125, is often used as a serum diagnostic and prognostic biomarker for PDAC and other cancers, in combination with other well-known markers such as CA19-9 and carcinoembryonic antigen (CEA). MUC16 is the largest membrane-associated mucin, constituted by a short intracellular tail, a transmembrane domain, and a large extracellular region containing several tandem repeats, multiple SEA functional groups and a heavily glycosylated N-terminal domain 122,123.

MUC16 is normally found on epithelial cells of the respiratory tract, female reproductive tissues, cornea and conjunctiva, while overexpression has been observed in various types of cancer. In PDAC, expression of MUC16 has been reported in over 80% of 200 cases, whereas it is absent in the vast majority of normal pancreatic samples (112/115) 87. In immunohistochemical analysis of patient-derived tissues, the expression of MUC16 is mostly limited to the cancer cells and not to the surrounding stroma 88. Although MUC16 expression is shown to increase as PDAC advances, levels in precursor lesions were found to be relatively low, suggesting this mucin might not be the best target for the early diagnosis 87,88. Nonetheless, MUC16 expression was shown to be strongly correlated with short patient survival, with particularly high expression at metastatic sites, indicating that this target plays a significant role in PDAC pathophysiology 40,87.

The role of MUC16 in PDAC involves the interaction of this mucin with several proteins. One of the most reported interactions is with mesothelin, which activates matrix metalloproteinases, resulting in increased cell motility and invasion 47. MUC16 is also thought to be involved in cell invasion and metastasis, with a potential role of the aberrant glycans at its surface in MUC16-galectin-3 mediated metastasis, and also through interaction of MUC16 with focal adhesin kinase (FAK). The latter possibly can cause changes in the tumor cell adhesion and invasion, as a result of FAK-mediated Akt and ERK/MAPK signaling 47. MUC16 upregulation was also linked to increased cell proliferation and tumor size*,* indicating that this mucin might induce cell proliferation, possibly through interaction with FAK 47. Moreover, MUC16 might play a role in immune evasion, as its expression has been correlated with upregulation of immunosuppressive regulatory T cells 48. Furthermore, MUC16 mutation has been correlated with extremely long survival and selective loss of high quality and MUC16 neoantigenic clones on metastatic progression has been found, suggesting neoantigen immunoediting 124.

As an established biomarker in PDAC, MUC16 has potential as diagnostic and therapeutic target. Of the developed treatments, however, only the monoclonal antibody oregovomab was evaluated in clinical trials, also in combination with chemo- and radiotherapy in a phase II study (NCT01959672). As expression of MUC16 has been linked to T-cell specific immunosuppression, one of the goals of this study was to evaluate the effects of oregovomab on the immune response. The study reported the development of MUC16-specific CD8 T-lymphocytes in some of the patients 48. However, these results are not conclusive as the study was limited by a small sample size, and therefore further studies are needed to confirm these findings.

MUC16 is currently, being investigated in preclinical studies for imaging to aid in the removal of PDAC tumor during surgery. For instance, the fluorescent MUC16-targeted antibody probe AR9.6-IRDye800 was recently tested in an orthotopic xenograft mouse model. The probe was able to achieve tumor visualization with low background, indicating the potential of MUC16 for imaging 48. An overview of the clinical trials targeting MUC16 is summarized in Table 3.

## Discussion

PDAC is a complex disease with high mortality rate. The lack of effective treatment is due to high percentage of late-stage diagnosis, as well as biological aggressiveness and therapeutic resistance. Therefore, it is imperative to develop strategies for early diagnosis of PDAC and more effective and better-tolerated therapeutic options. While many targets have been investigated, only few have shown encouraging results. In this review we discussed a number of transmembrane proteins as promising targets in PDAC, based on studies published in the last 10 years. The selection of the targets was based on favorable features, namely high expression in a large percentage of PDAC cases, low expression in healthy tissues, as well as promising preclinical or clinical results.

Transmembrane proteins containing extracellular domains, can be excellent targets for the delivery of the therapeutic agents and (early) imaging. Various examples, especially in nuclear medicine, are proven successful for other clinical indications. In PDAC the expression of many transmembrane proteins is significantly increased, making them viable candidates for the development of specific imaging strategies and effective treatments with low side effects. Importantly, large variation between patients in terms of the protein expression level has been reported, resulting in inconsistent patterns of protein expression. Among the large number of proteins overexpressed in PDAC, only a few have shown favorable characteristics for effective targeting and imaging with promising preclinical and clinical results. Due to the large patient-to-patient variability, it is important to focus on targets that are overexpressed in the majority of PDAC patients. The majority of the targets described in this review have shown overexpression in a large percentage of cases, although for some targets there is large variation and expression in PDAC has not been as extensively studied for all targets.

Mesothelin and PDGFRβ have shown *de novo* expression in up to 100% of cases, showing strong expression in 80-85% and 49% of the cases, respectively. Importantly, mesothelin and PDGFRβ are not expressed in normal pancreatic tissue and therefore can be ideal targets for therapy and imaging. In particular for PDGFRβ, which has also been described to be expressed by tumor stroma, further studies are needed to assess the consequence of this for diagnostic or therapeutic purposes. To our understanding, even though ASPH, GPRC5A, HER2, integrin αvβ6 and mucins are expressed to some extend in normal tissues, their expression in normal pancreatic tissue has not been reported.

Expressions of EGFR and HER2 have shown a large variation in PDAC patients. In fact, while some studies have reported EGFR expression in nearly 90% of cases, others have reported much lower percentage (30%) 4,62. Similarly, HER2 expression in PDAC varies from 0-82% 65. The possible explanation for the differences might be related to the different scoring methods or interpatient variation, making it difficult to draw firm conclusions about the actual percentage of expression in PDAC. Thus, patient eligibility for EGFR or HER2 targeted therapies may need to be determined in a more personalized manner, for instance by assessing EGFR or HER2 expression by immunohistochemistry (IHC) on biopsies.

In addition to the therapeutic potential of the targets, overexpression of these proteins can be exploited for development of novel imaging strategies for (early) diagnosis and follow-up of PDAC. Early PDAC diagnosis has proven to be challenging, contributing to poor survival of patients. Among the targets discussed, currently only integrin αvβ6 is assessed for imaging purposes in clinical trials (NCT03164486, NCT02683824). However, other targets such as claudin 4, EGFR, mesothelin, MUC4 and MUC16 have shown promising results for PDAC imaging *in vivo* 48,75,97,98,110,111,117,118,121. Although this has not yet been confirmed, proteins such as ASPH and PDGFRβ that are not detectable in healthy pancreas could also be potential targets for PDAC imaging.

Besides overexpression of the targets on tumor cells compared to healthy pancreas, absence of expression in other healthy tissues is also important for therapy. Therefore, identification of targets with high expression in PDAC and low or preferably no expression in healthy tissues is essential for the development of effective treatment with low side effects. In this regard, mesothelin and ASPH have shown the most promising characteristics. The expression of mesothelin is limited to mesothelial cells in the pleura, pericardium and peritoneum, and also according to the preclinical studies, mesothelin does not play a role in normal cell processes and development 38,81,82,114. Similarly, ASPH is not expressed in healthy pancreatic tissue nor is it in many healthy tissues. Importantly, ASPH is an oncofetal protein which remains inactive after the embryonic development, further encouraging targeting of this protein in PDAC.

In spite of the expression of EGFR, claudin 4, GPRC5A, HER2, integrin αvβ6 and mucins in several normal tissues, the expression level is significantly higher in PDAC compared to other tissues, although the exact difference in expression levels has not been reported 61,74,125. Nonetheless, lower expression in normal tissues would likely reduce side effects compared to the effects on tumor cells. To further reduce the side effects on the normal tissues, therapeutic agents could be applied locally at the tumor site. Last but not least, the elevated interstitial fluid pressure particularly present in PDAC appears to hinder efficient drug delivery. Combination of targeted therapies with local ablation therapies, such as radiofrequency (RFA), microwave (MWA), cryoablation (CA), and irreversible electroporation, could help to overcome this.

In conclusion, the development of effective treatments has been challenging due to the complexity of PDAC, and novel diagnostic and therapeutic strategies are needed. Many clinical studies have been conducted and more trials are still ongoing investigating the potential of therapeutic agents as monotherapy or in combination. Transmembrane proteins described in this review could serve as potential targets for diagnosis, and also to improve treatment efficacy and quality of patients' life.

## Figures and Tables

**Table 1:** Summary of the characteristics of the 11 selected transmembrane proteins described in this review for diagnosis and/or treatment of PDAC

Target	Molecular weight (kDa)	Ligands	Protein type	Role	Reference
ASPH	~86	No ligand	α-ketoglutarate-dependent dioxygenase	Proliferation, migration, invasion, hydroxylation of EGF-like domain containing proteins	9
EGFR	~134	EGF, TGFA, HBEGF, BTC, AREG, EREG, EPGN	Tyrosine kinase receptor	Proliferation, metastasis, drug resistance, survival	11,20,21
HER2	~185	No ligand	Tyrosine kinase receptor	Proliferation, migration, invasion, survival	22-24
PDGFRβ	~90	PDGF	Tyrosine kinase receptor	proliferation, migration, invasion, angiogenesis, survival	25-27
GPRC5A	~40	No ligand	G protein-coupled receptor	Proliferation, invasion, metastasis, chemoresistance	28-30
Claudin 4	~22	CPE	Adhesion protein	Tumor progression, invasion, chemoresistance	31-34
Integrin αvβ6	~114	Fibronectin,Vitronectin, Tenascin-C, TGF-β1	Adhesion glycoprotein	Proliferation, metastasis, invasion, immune evasion	35,36
Mesothelin	~40	No ligand	GPI-anchored surface protein	Proliferation, metastasis, angiogenesis, chemoresistance, activation of matrix metalloproteinases by interaction with MUC16	37-39
MUC4	550-590	No ligand	Highly glycosylated tight junction protein	Proliferation, metastasis, immune evasion, chemoresistance	40-42
MUC13	~175	No ligand	Highly glycosylated protein	Activation of pro-oncogenic proteins, metastasis, down regulation of p53	43-46
MUC16	3000-5000	No ligand	Highly glycosylated protein	Proliferation, activation of matrix metalloproteinases by interaction with mesothelin, metastasis, immune evasion	47-49

ASPH: Aspartate-β-hydroxylase; EGFR: epidermal growth factor receptor; EGF: epidermal growth factor; TGFA: transforming growth factor-alpha; HBEGF: heparin-binding EGF-like growth factor; BTC: betacellulin; AREG: amphiregulin; EREG: epiregulin; EPGN: epigen; HER2: human epidermal growth factor receptor 2; PDGFRβ: platelet derived growth factor receptor beta; PDGF: platelet derived growth factor; GPRC5A: G protein-coupled receptor family C member 5 group A; CPE: *Clostridium perfringens* enterotoxin; TGF-β1: transforming growth factor; HER2: human epidermal growth factor receptor 2; GPI: glycosylphosphatidylinositol.

**Table 2:** Selected transmembrane proteins described for the diagnosis or treatment of PDAC

Target	Expression in PDAC	Percentage of expression	Stage of disease	Normal tissue expression	Reference
ASPH	Tumor cells	97%	Lesions<4	Placental trophoblasts	9,10,60
EGFR	Tumor cells, endothelial cells of the microenvironment	30-90%	Lesion-4	Gastrointestinal tract, pancreas, skin, brain, vasculature, heart, lungs, kidneys, liver, reproductive organs	4,61-64
HER2	Tumor cells	0-82%	1-4	Adrenal gland, liver, skin, brain, heart	65-68
PDGFRβ	Tumor cells, stroma	~100% positive, 49% strong	1<4	Reproductive tissues, kidneys	25,26,69-71
GPRC5A	Tumor cells	82%	1<4	Lung (high), intestines, kidneys, thyroid, bladder (low)	28-30,72
Claudin 4	Tumor cells	70-97%	Lesions-4	Lungs, stomach, liver, intestines, kidneys, pancreas	73-76
Integrin αvβ6	Tumor cells (mainly), fibroblasts, vessels (rarely)	~90%	Lesions<4	esophagus, stomach, intestines, liver	35,36,77-79
Mesothelin	Tumor cells	100% positive, 80-85% strong	Lesions-4	Pleura, pericardium and peritoneum	37,80-83
MUC4	Tumor cells	70-95%	Lesions<4	Airway epithelia, body fluids	42,84-86
MUC13	Tumor cells	95%	Lesions<4	Trachea, stomach, intestines, kidneys	43-45
MUC16	Tumor cells	82%	1<4	Airway epithelia, female reproductive tissues, cornea, conjunctiva	41,87,88

ASPH: Aspartate-β-hydroxylase; EGFR: epidermal growth factor receptor; GPRC5A: G protein-coupled receptor family C member 5 group A; HER2: human epidermal growth factor receptor 2; PDGFRβ: platelet derived growth factor receptor beta. Stage of disease indicates in which phase overexpression of transmembrane proteins is observed. In some targets the expression remains steady over the disease progression, i.e. from lesion to stage 4 (lesion-4) or from stage 1 to stage 4 (1-4), while increasing in other targets i.e. from lesion to stage 4 (lesion<4) or from stage 1 to stage 4 (1<4).

**Table 3:** Summary of the identified clinical trials targeting the transmembrane proteins for the diagnosis and treatment of PDAC

Target	Trial number	Phase	Intervention	# of patients
EGFR	NCT04137536	1	Anti-EGFR-BATs	25
	NCT00243854	1	Neoadjuvant hypofractioned radiotherapy + gemcitabine + erlotinib	8
	NCT00536614	2	Gemcitabine/Cisplatin +/- Cetuximab	86
	NCT03269526	1/2	Anti-CD3 x anti-EGFR-BATs	22
	NCT02620865	1/2	Anti-CD3 x anti-EGFR-BATs + aldesleukin + sargramostim	2
	NCT00042939	2	Cetuximab + irinotecan + docetaxel	94
	NCT00424827	2	Cetuximab + gemcitabine + 5-FU + external beam radiotherapy	11
	NCT01077986	1/2	Cetuximab + capecitabine + everolimus	35
	NCT01420874	1	Anti-EGFR-BATs + FOLFOX6	30
	NCT00040183	3	Erlotinib + gemcitabine	569
	NCT00862524	1/2	ARRY-334543 + gemcitabine	20
	NCT00234416	1/2	Gefitinib + gemcitabine	45
	NCT01505413	2	Erlotinib + gemcitabine + oxaliplatin	33
	NCT01210911	2	Erlotinib + gemcitabine +/- metformin	120
	NCT02451553	1	Afatinib + capecitabine	48
	NCT00260364	1/2	Gemcitabine + capecitabine + erlotinib + bevacizumab	44
	NCT01693419	2	Erlotinib + gemcitabine + S-1	37
	NCT04464967	1/2	SNK01 + trastuzumab; SNK01 + cetuximab	154
	NCT00026338	3	Gemcitabine +/- erlotinib	569
	NCT04429542	1	BCA101 +/- pembrolizumab	292
	NCT00225784	2	Cetuximab + gemcitabine + radiotherapy	37
	NCT00480584	1	Erlotinib + gemcitabine + capecitabine	20
	NCT00397384	1	Cetuximab + erlotinib	43
	NCT00383149	2	Cetuximab + ixabepilone	58
	NCT00622674	1	Cetuximab + bortezomib	37
	NCT03319459	1	FATE-NK100; FATE-NK100 + cetuximab; FATE-NK100 + trastuzumab	100
	NCT00962312	2	Lapatinib + capecitabine	9
	NCT00075686	3	Gemcitabine +/- cetuximab	766
	NCT01222689	2	Erlotinib + selumetinib	46
	NCT03989115	1/2	RMC-4630 + Osimertinib; RMC-4630 + cometinib	168
	NCT03878524	1	Several drug combinations including afatinib,dacomitinib, erlotinib, imatinib, trastuzumab	40
	NCT02465060	2	Several drug combinations including afatinib, dacomitinib, erlotinib, imatinib, trastuzumab	6452
HER2	NCT00862524	1/2	ARRY-334543 + gemcitabine	20
	NCT04464967	1/2	SNK01 + trastuzumab or SNK01 + cetuximab	154
	NCT02999672	2	Trastuzumab	20
	NCT02451553	1	Afatinib + capecitabine	48
	NCT03602079	1/2	A166	82
	NCT04482309	2	Trastuzumab deruxtecan	280
	NCT02912949	1/2	Zenocutuzumab	250
	NCT03586869	1/2	ETBX-021 + several other drugs	173
	NCT00005926	2	Herceptin(trastuzumab) + gemcitabine + radiotherapy	50
	NCT00034281	1	TAX-165	16
	NCT00004074	1	Trastuzumab + IL-12	15
	NCT01384253	1	²¹²Pb-TCMC-Trastuzumab + trastuzumab	18
	NCT00962312	2	Lapatinib + capecitabine	9
	NCT03319459	1	FATE-NK100; FATE-NK100 + cetuximab; FATE-NK100 + trastuzumab	100
	NCT04319757	1	ACE1702 (anti-HER2 oNK cells) + cyclophosphamide + fludarabine	24
	NCT03425773	1	BVAC-B	8
	NCT02465060	2	Several drug combinations including afatinib, dacomitinib, erlotinib, imatinib, trastuzumab	6452
Integrin αvβ6	NCT02683824	1	[18F]-R01-MG-F2 for PET/CT and PET/MRI	25
	NCT04285996	NA*	[18F]FBA-A20FMDV2 for PET	12
	NCT03023722	2	Anetumab ravtansine	18
	NCT03638193	NA*	CART-meso cells	10
	NCT01897415	1	Mesothelin targeted CAR-T	16
	NCT03816358	1/2	Anetumab ravtansine + nivolumab; +ipilimumab + nivolumab; + gemcitabine + nivlumab	64
	NCT03122106	1	Personalized neoantigen DNA vaccine	10
Mesothelin	NCT00066651	1	SS1(dsFv)-PE38 immunotoxin	15
	NCT00006981	1	SS1(dsFv)-PE38 immunotoxin	30
	NCT00325494	1	MORAb-009(amatuximab)	24
	NCT03956056	1	Neoantigen peptide vaccine + poly ICLC	15
	NCT01521325	1	Amatuximab	6
	NCT03323944	1	huCART-meso cells	18
	NCT03102320	1	Antumab ravtansine +/- gemcitabine/cisplatin	173
	NCT04034238	1	LMB-100 + tofacitinib	45
MUC16	NCT01959672	2	Oregovomab + gemcitabine + leucovorin + 5-FU + nelfinavir + radiotherapy	11
PDGFRβ	NCT03766295	3	Masitinib + gemcitabine; gemcitabine	377
	NCT00789633	3	Masitinib + gemcitabine; gemcitabine	353
	NCT00967603	2	Sunitinib	56
	NCT00673504	2	Sunitinib + gemcitabine; gemcitabine	105
	NCT00397787	2	Sunitinib	64
	NCT03878524	1	Several drug combinations including sunitinib, dacomitinib, erlotinib, imatinib, trastuzumab	40
	NCT02465060	2	Several drug combinations including sunitinib, dacomitinib, erlotinib, imatinib, trastuzumab	6452
	NCT00462553	1	Sunitinib + gemcitabine	37
	NCT00703638	1	Sorafenib + cisplatin + pemetrexed	16
	NCT00837876	2	Sorafenib + erlotinib	37
	NCT00789763	1	Sorafenib + gemcitabine + radiotherapy	12
	NCT00696696	2	Sorafenib + erlotinib + gemcitabine	45
	NCT00114244	2	Sorafenib; sorafenib + gemcitabine	52
	NCT00375310	1	Sorafenib + gemcitabine + radiotherapy	27
	NCT00095966	2	Sorafenib + gemcitabine	35
	NCT02349867	1	Sorafenib + gemcitabine + vorinostat + radiotherapy	23
	NCT00981162	1	Sorafenib + everolimus	12
	NCT01497392	1	Dovitinib + gemcitabine + capecitabine	26

*NA: Not Applicable; EGFR: epidermal growth factor receptor; HER2: human epidermal growth factor receptor 2; PDGFRβ: platelet derived growth factor receptor beta.
